# Diversity and Dynamics of Active Small Microbial Eukaryotes in the Anoxic Zone of a Freshwater Meromictic Lake (Pavin, France)

**DOI:** 10.3389/fmicb.2016.00130

**Published:** 2016-02-10

**Authors:** Cécile Lepère, Isabelle Domaizon, Mylène Hugoni, Agnès Vellet, Didier Debroas

**Affiliations:** ^1^Laboratoire “Microorganismes: Génome et Environnement”, Clermont Université, Université Blaise PascalClermont-Ferrand, France; ^2^Centre National de la Recherche Scientifique, UMR 6023, LMGEAubière, France; ^3^Institut National de la Recherche Agronomique, UMR 42 CARRTELThonon-les-Bains, France; ^4^Université Savoie MontBlancChambéry, France

**Keywords:** microbial eukaryotes, anoxia, lakes, active biosphere, 454 pyrosequencing

## Abstract

Microbial eukaryotes play a crucial role in ecosystem functioning and oxygen is considered to be one of the strongest barriers against their local dispersal. However, diversity of microbial eukaryotes in freshwater habitats with oxygen gradients has previously received very little attention. We applied high-throughput sequencing (V4 region of the 18S rRNA gene) in conjunction with quantitative PCR (DNA and RNA) and fluorescent *in situ* hybridization (FISH) analyses, to provide an unique spatio-temporal analysis of microbial eukaryotes diversity and potential activity in a meromictic freshwater lake (lake Pavin). This study revealed a high genetic diversity of unicellular eukaryotes in the permanent anoxic zone of lake Pavin and allowed the discrimination of active vs. inactive components. Forty-two percent of the OTUs (Operational Taxonomic Units) are exclusively present in the monimolimnion, where Alveolata (Ciliophora and Dinophyceae) and Fungi (Dikarya and Chytrids) are the most active phyla and are probably represented by species capable of anaerobic metabolism. Pigmented eukaryotes (Haptophyceae and Chlorophyceae) are also present and active in this zone, which opens up questions regarding their metabolism.

## Introduction

Life without oxygen is common on earth. Anoxia can be temporary or permanent but oxygen is always a strong shaping force and it is a dominating factor determining functional interactions and the spatial structure of many microbial communities. Very few studies have focused on microbial eukaryote survival and distribution in anoxic waters or low oxygen environments, especially in comparison with what we already know about bacteria, and most of these studies have been conducted in marine ecosystems (Fenchel and Finlay, 2008). However, these works have revealed substantially rich species diversity (Dawson and Pace, 2002; Behnke et al., 2006; Stoeck et al., 2006; Zuendorf et al., 2006; Charvet et al., 2012; Oikonomou et al., 2014a). Sequence analysis of both nuclear (18S) and plastid (16S) ribosomal RNA genes revealed a great variety of heterotrophic unicellular eukaryotes as well as phytoplankton in anoxic marine samples (López-García et al., 2001; Dawson and Pace, 2002; Stoeck et al., 2009) and in freshwaters (Oikonomou et al., 2014a). These studies show that the most represented sequences are affiliated to Alveolata (Ciliates, Dinoflagellates, uncultured Alveolates, and Perkinsozoa) and Stramenopiles [Diatoms, Chrysophytes and MAST (MArine STramenopiles)] (Behnke et al., 2010; Charvet et al., 2012; Rocke et al., 2012; Triadó-Margarit and Casamayor, 2015). Survival in anoxic habitats requires a means of anaerobic energy metabolism, which is far more varied in prokaryotes (Amend and Shock, 2001) than in eukaryotes (Müller et al., 2012). The presence of unicellular eukaryotes in anoxic environments can therefore be surprising, especially for the putative photosynthetic ones. However, anoxia is not exceptional for photosynthetic algae, but often transitory. Photosynthetic algae, such as Chlorophyta and diatoms, have also been discovered in a variety of chemically extreme environments such as hypersaline waters, natural rock acid drainages, acid mine drainages, and sulfidic lakes (Zettler et al., 2002; Alexander et al., 2009; Brake and Hasiotis, 2010; Triadó-Margarit and Casamayor, 2015). The metabolic responses of algae to anoxia have been the subject of examination for some time, however, only a few green algae, such as *Chlamydomonas reinhardtii, Chlamydomonas moewusii, Chlorogonoium elongatum, Chlorella fusca*, and *Scenedesmus* D3 have been studied to date (Atteia et al., 2013).

Among freshwater environments, meromictic lakes, which present an oxygen gradient along the water column and a permanent anoxic zone, provide ideal systems to study microbial communities associated to biogeochemical processes in stratified water bodies. High physical stability of the water masses results in relatively constant vertical physico-chemical gradients, which may provide a variety of niches for microbial growth and differentiation (Pouliot et al., 2009). To our knowledge only two studies targeted the permanent anoxic zone (Charvet et al., 2012; Oikonomou et al., 2014a) but failed to explore the potential activity of eukaryotes by studying, for instance, the community structure at both the DNA and RNA level (Campbell et al., 2011; Logares et al., 2014; Hugoni et al., 2015). In general, studies focusing on “extreme environment” utilize SSU rDNA clone library, do not approach sampling saturation, and underestimate the diversity of eukaryotic microbes in an environmental sample. The development of high-throughput sequencing technologies reduces these limitations and provides a much more realistic estimate of their total diversity and community structure (Bik et al., 2012).

In the present study, we focused our analysis on microbial eukaryotes which are of crucial importance when it comes to energy and carbon flow (Finlay and Esteban, 1998; Sherr and Sherr, 2002). This assemblage includes diverse phototrophic, mixotrophic, and heterotrophic cells, which play fundamental roles as primary producers, bacterial grazers, and parasites (Stockner and Antia, 1986; Caron et al., 1999; Guillou et al., 2008; Lepère et al., 2008; Hartmann et al., 2013). Therefore, the ecosystem functioning is impacted by the taxonomic composition of microbial eukaryote communities in specific habitats. We used high-throughput amplicon sequencing of the V4 region of the 18S rRNA gene, in combination with quantitative PCR (DNA and RNA) and Fluorescent *In Situ* Hybridization (FISH) analyses to provide the first in-depth molecular analysis of unicellular eukaryotic community diversity and potential activity in the permanent anoxic zone of a meromictic freshwater lake (Pavin), during a 1-year study.

## Methods

### Study site

Lake Pavin, located at 45°55 N and 2°54 E, is the youngest volcano crater lake in the French Massif Central (6000 years BP). Lake Pavin is a circular lake with an area of 0.44 km^2^ and a maximum depth of 92 m, at an altitude of 1197 m above sea level. It is fed by atmospheric precipitations and numerous superficial and sub-lacustrian springs (Viollier et al., 1997). Lake Pavin is a meromictic lake characterized by the presence of two permanent stratified layers. The upper layer (mixolimnion) extends from the surface to 60 m depth and is affected by mixing during fall and spring. The deepest layer (monimolimnion) extends from 60 to 92 m depth and includes the chemocline (60–70 m) (Lehours et al., 2005).

### Sampling procedures

From March to December 2011 water samples were collected monthly in the oxygenated and anoxic zone, respectively at 2 (mixolimnion) and 80 m (monimolimnion), using a Van Dorn bottle at a permanent station located at the deepest zone of the water column. The water temperature, dissolved oxygen content, phosphorus (P-PO_4_), nitrate (N-NO_3_), and ammonia (N-NH_4_) concentrations at 80 m can be found in Supplementary Table 1. Because of the strong concentrations of Iron II in the monimolimnion of lake Pavin, samples for FISH experiments were filtered on-site with CO_2_ flushed materials in order to limit the iron oxidation.

### Flow cytometry

Samples were analyzed using a FACSCalibur flow cytometer (Becton Dickinson, San Jose, CA) equipped with a laser emitting at 488 nm and a 70-μm nozzle. Emitted light was collected through the following set of filters: 488/10 band pass (BP) for side scatter, 576/26 BP for orange fluorescence (Phycoerythrin, FL2), and 655 long pass for red fluorescence (Chlorophyll, FL3). Signal detection was triggered on the chlorophyll fluorescence. Samples were run for 2 min at a flow rate of 40 μL/min to estimate pigmented picoplankton abundances.

### Nucleic acids extraction

A water sample of 300 mL was added with an equal volume of RNA Later [ammonia sulfate 7.93 M, sodium citrate 0.025 M, EDTA 0.02 M qsp 1.5 L of RNAse free water (pH 5.2)], pre-filtered through 5 μm pore-size polycarbonate filters (Millipore) and then filtrated on 0.2 μm pore-size (pressure < 10 kPa) polycarbonate filters (Millipore) before storage at −80°C until nucleic acids extraction. It is well known that whatever the aquatic ecosystem studied, prefiltration allows some cells that are larger than their nominal pore sizes to pass through and can lead to the retention of smaller cells if the filters are clogged. However, after comparing nonfiltered and filtered fractions, in a previous study, we detected a slight decrease in total abundance (10–15%) but no modification of diversity inferred by morphological inspection (Lefranc et al., 2005). The nucleic acids extraction method was modified from Hugoni et al. (2013b) using a combination of mechanical and enzymatic cell lysis, followed by extraction using the AllPrep DNA/RNA kit (Qiagen, Valencia, CA). RNA samples were tested for the presence of contaminating genomic DNA using PCR and then reverse transcribed with random primers using SuperScript^®^ VILO (Invitrogen).

### Pyrosequencing, cleaning procedures, clustering, and taxonomic affiliation

Amplification of the V4 region of the 18S rRNA genes (rDNA) and 18S rRNA was performed using the eukaryotic primers Ek-NSF573 (CGCGGTAATTCCAGCTCCA) and Ek-NSR951 (TTGGYRAATGCTTTCGC) (Mangot et al., 2012). Pyrosequencing was achieved by the GINA Platform (Clermont-Ferrand, France), using a Roche 454 GS-FLX system with titanium chemistry. Pyrosequencing data for both 18S rDNA and 18S rRNA datasets represented 304,315 raw sequences (total for 2 and 80 m depths). The cleaning procedure of the reads is summarized in Supplementary Figure 1. Briefly, cleaning procedures consisted in the elimination of sequences presenting ambiguous bases “N,” a quality score < 25, length shorter 200 bp, with a mismatch in the forward primer and chimeras (Edgar, 2010). The remaining sequences were clustered at a 95% similarity threshold as suggested by Debroas et al. (2015) with UCLUST (Edgar, 2010) and representative sequence for each OTU were aligned (Eddy, 1998) and inserted in phylogenetic trees with Fasttree (Price et al., 2010) for taxonomic annotation. After the cleaning of 454 raw data and discarding the singletons and OTUs without rDNA reads, we defined 7737 OTUs for the monimolimnion and 6883 for the mixolimnion. This process was automated by PANAM that also computed richness and diversity indexes, Chao1 and Shannon respectively (http://code.google.com/p/panam-phylogenetic-annotation/downloads/list) (Taib et al., 2013). We also performed a hierarchical cluster analysis based on Bray Curtis distance in order to compare the structure of eukaryotic communities for all DNA samples between 2 and 80 m (XLSTAT 2015 4.01). The monimolimnion (80 m depth) dataset contained a total of 44,000 sequences for the 18S rDNA dataset and 10,324 sequences for the 18S rRNA dataset (the latter was obtained for one date, 05/07/2011). The data were normalized to have an equal number of rRNA and rDNA reads per sample in order to infer on the activity of eukaryotic taxa (rRNA:rDNA ratio). The data have been deposited in the Dryad Digital Repository^1^.

### Quantitative PCR analysis

The qPCR protocol was modified from Hugoni et al. (2013a) and used to quantify eukaryotic 18S rRNA transcripts. Amplification of the V4 region of the 18S rRNA was performed using the primers described above. The reaction mixture (25 μL) contained MESA GREEN qPCR MasterMix Plus for SYBR Assay^®^ (1X, Eurogentec) added with 0.8 μg of BSA, 0.7 μM of primers and ultra-pure sterile water. One microliter of nucleic acids was added to 24 μL of mix in each well. All qPCR reactions were performed in triplicate and PCR cycling conditions were 10 min at 94°C (initial denaturation) followed by 45 cycles at 94°C (30 s), 60°C (30 s), and 72°C (45 s) using a Mastercycler^®^ep realplex real-time PCR system. Standard curves were generated from a mix of clones that were representative of the environment studied. All reactions were performed with standard curves spanning from 10^1^ to 10^8^ copies per μL. The PCR efficiency was 74% and *r*^2^ = 0.98.

### TSA-FISH (tyramide signal amplification-fluorescent *in situ* hybridization)

Water samples were prefiltered through 5 μm-pore-sized polycarbonate filters (Millipore, Molsheim, France) at a very low vacuum to prevent cell damage and collected on 0.2 μm pore-size polycarbonate filters (Millipore). Filtrations were realized under CO_2_ flux. The filters were preserved by dehydration in an ethanol series (50, 80, and 100% for 3 min each) and stored at −20°C in the dark until analysis.

The probe used in this study were EUK1209 (Giovannoni et al., 1988), CHLO02 (Simon et al., 2000), CRYPT 13 (Lepère et al., 2008), PRYM02 (Simon et al., 1995), CHRYSO_01 (Mangot et al., 2009), MY1574 (Baschien et al., 2008) targeting, respectively, total eukaryotes, Chlorophyceae, Cryptophyceae, Prymnesiophyceae, Chrysophyceae, and Fungi. TSA-FISH was performed as described in Lepère et al. (2010). Briefly, the hybridization filters were covered with a hybridization buffer (40% deionized formamide, 0.9 M NaCl, 20 mM Tris-HCl [pH 7.5], 0.01% sodium dodecyl sulfate, 10% blocking reagent [Roche Diagnostics/Boehringer]) and oligonucleotide probes labeled with horseradish peroxidase. The mixture was left to hybridize at 35°C for 3 h. After two successive 20-min rinses at 37°C in a wash buffer (56 mM NaCl, 5 mM EDTA, 0.01% sodium dodecyl sulfate, 20 mM Tris-HCl [pH 7.5]), samples were equilibrated in TNT buffer (7% Tween 20, 150 mM NaCl, 100 mM Tris-HCl [pH 7.5]) at room temperature for 15 min. Tyramide amplification was performed for 30 min at room temperature in the dark in TSA mix, a mixture (1:1) of 40% dextran sulfate (Sigma-Aldrich) and 1X amplification diluent (Perkin-Elmer LAS), which provide enhanced sensitivity, to which is added fluorescein isothiocyanate coupled with tyramide (1X; Perkin-Elmer LAS) (1:50). Filters were then transferred through two successive 5-ml TNT buffer baths at 55°C for 20 min each to stop the enzymatic reaction and remove the dextran sulfate. Filters were mounted in a mixture of antifading oil AF1 (Citifluor, Biovalley, Conches, France) containing 10 μg mL^−1^ of propidium iodide which is a red-fluorescent nuclear counterstain (Sigma-Aldrich). Hybridized cells were examined under a Zeiss Axiovert 200 M inverted and epifluorescence microscope (Carl Zeiss, Jena, Germany) equipped with an HBO 100 W Hg vapor lamp at ¥ 100 magnification and Confocal (laser scanning) images were acquired with a Leica SP2 and the Leica confocal software carrying four different lasers allowing to see the two fluorochromes used here in combination.

## Results

### Is the microbial eukaryote community structure different between mixolimnion and monimolimnion?

The community structure of microbial eukaryotes was analyzed at 2 m (mixolimnion) and 80 m (monimolimnion) in order to detect potential specificities related to the anoxic zone. Total eukaryotic abundance was been measured by TSA-FISH at 2 and 80 m during the sampling period (March to December 2011). If major variations are recorded at 2 m depth (from 42,526 to 2639 cell ml^−1^) according to the seasons, the abundances at 80 m are lower with an average of 1868 cell.ml^−1^ (68–11,067 cell.ml^−1^) (Supplementary Figure 2). The diversity and richness are not significantly different between the two depths studied. SChao1 (calculated from 18S rDNA dataset) showed an annual average of 147 and 167 for 2 m and 80 m, respectively, whereas the Shannon index average is 5 and 4.5 at 2 and 80 m, respectively. When analysing the richness over the seasons, in spring and summer, the mixolimnion showed similar diversity values to the monimolimnion, however, in winter the global diversity at 80 m (SChao1 = 137) is almost twice less than at 2 m (SChao1 = 246) depth. In terms of major taxonomic groups at a low taxonomic resolution [i.e., level 2 of the classification used by EMBL (Nucleotide Sequence Database; http://www.ebi.ac.uk/embl/; http://www.ebi.ac.uk/ena)] there is only moderate variation among depths. However, at a finer taxonomic resolution (i.e., OTUs_95%_ level), the OTUs distribution between the 2 m and 80 m, allowed the identification of 42% OTUs exclusively present in the monimolimnion whereas only 12.5% of OTUs are present both in the mixolimnion and monimolimnion. The hierarchical clustering shows a clear separation between the two depths (Supplementary Figure 3). Moreover, significant differences in the microbial eukaryote composition were found between 2 and 80 m (ANOSIM *R* = 0.61, *P* < 0.0001). We hypothesized that the taxa specifically found in the monimolimnion were poorly referenced due to the low number of studies. Indeed, a Blast analysis showed that ~84% of the OTUs that have no close reference sequences available in public database (similarity < 85% to references found in Silva Database, Quast et al., 2013) are found in the monimolinion (Supplementary Table 2).

### Taxonomic composition and dynamics of microbial eukaryotes in the monimolimnion

**Figure 1:**
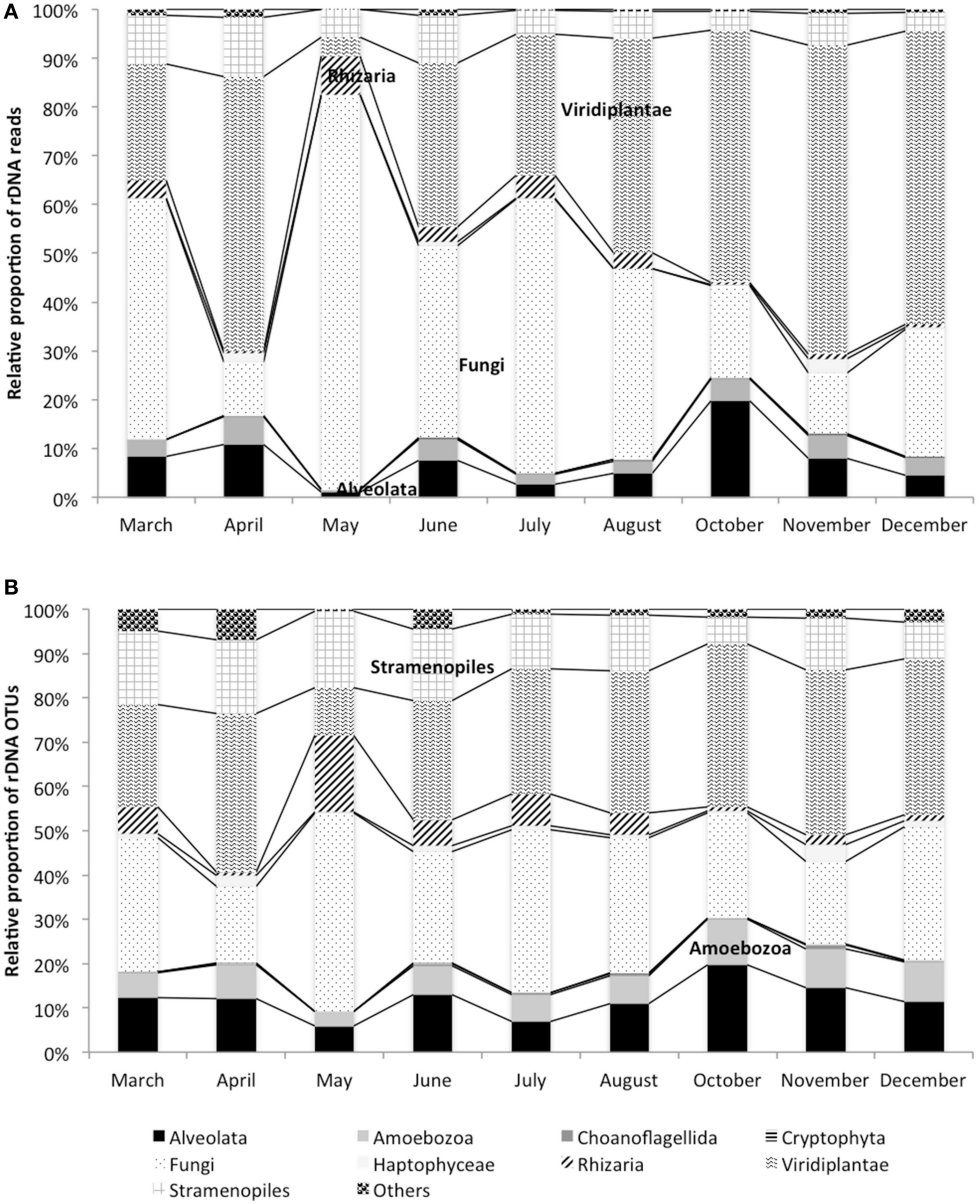
**Dynamics of rDNA reads (A) and rDNA OTUs (B) in the monimolimnion**.

18S rDNA dataset is dominated by Viridiplantae (41% of reads, 30% of OTUs), Fungi (33% of reads, 25% of OTUs), Stramenopiles (8% of reads, 14% of OTUs), and Alveolata (8% of reads, 12% of OTUs). These phyla are detected at all dates of the study period (Figure 1). Chlorophyta OTUs represent the majority of Viridiplantae (Supplementary Table 3). Their contribution along the year is stable except for April when their OTUs richness doubles (619 OTUs) compare to the average (250 OTUs), and May when the contribution drops to 38 OTUs (Figure 1 and Supplementary Figure 4). Among Fungi, Dikarya and Chrytrids are the most frequently encountered and are present at all dates (Figure 1 and Supplementary Table 3). Stramenopiles are dominated by Bacillariophyta, along the sampling period, but Bicosoecida and Chrysophyceae are also found. Whereas, Chrysophyceae are present at all dates, Bicosoecida are completely absent in October (Supplementary Figure 4). Among Alveolata, Dinophyceae, and ciliates are the most represented OTUs and are recovered along the sampling period. The 42% of OTUs exclusively present in the monimolimnion are affiliated to Fungi (Ascomycota, Basidiomycota, and Chytrids), Chlorophyta (Trebouxiophyceae, and Chlorophyceae), Haptophyta (Prymnesiales), Stramenopiles (Bacillariophyta and unclassified Stamenopiles), and Alveolata (Ciliates and unclassified Alveolata).

### Are microbial eukaryotes active in the monimolimnion?

**Figure 2:**
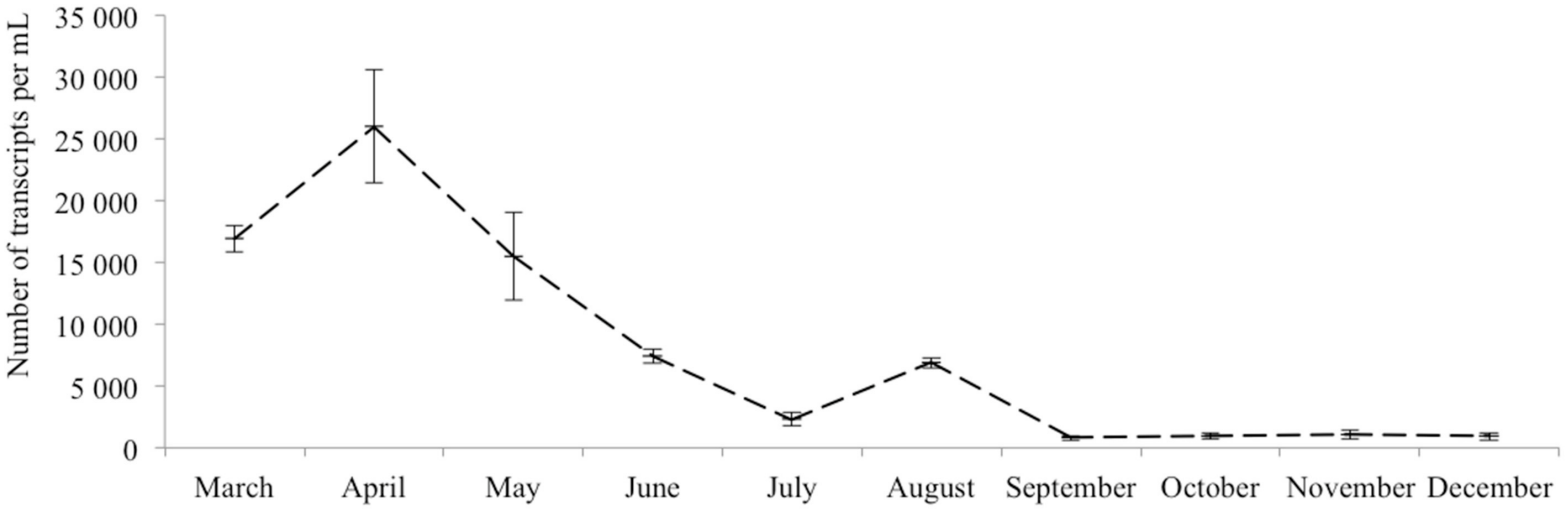
**18S rRNA transcripts abundance (qPCR) of active microbial eukaryotes in the monimolimnion**.

**Figure 3:**
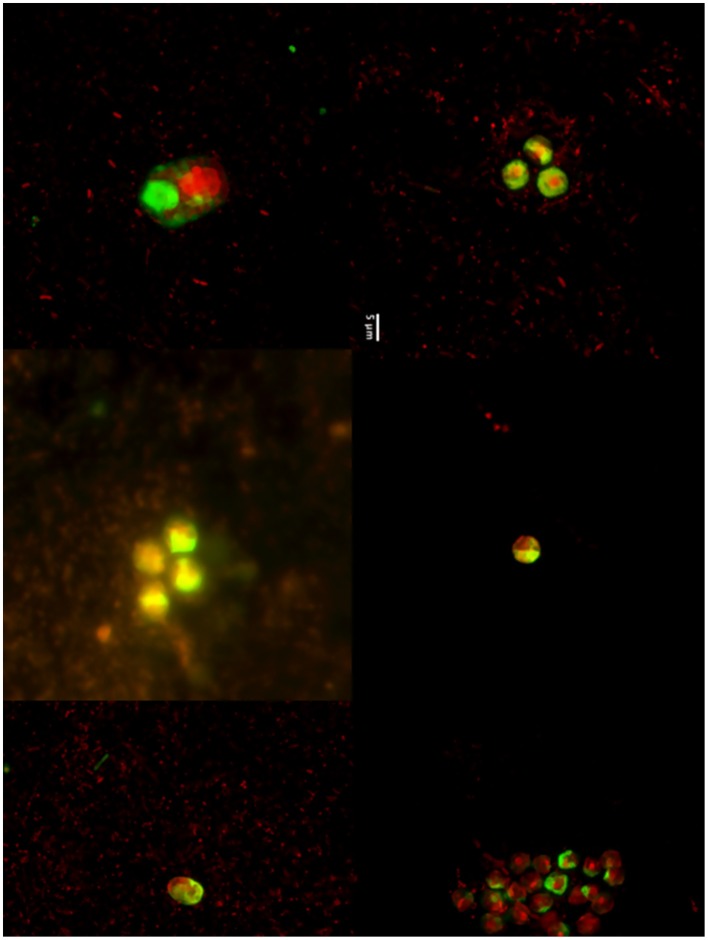
**TSA-FISH micrographs (from left to right) of microbial eukaryotes (EUK1209), Chlorophyceae (CHLO02), Prymnesiophyceae (PRYM02), Cryptophyceae (CRYPT 13), Chrysophyceae (CHRYSO_01), and Fungi (MY1574) in the monimolimnion**.

**Figure 4:**
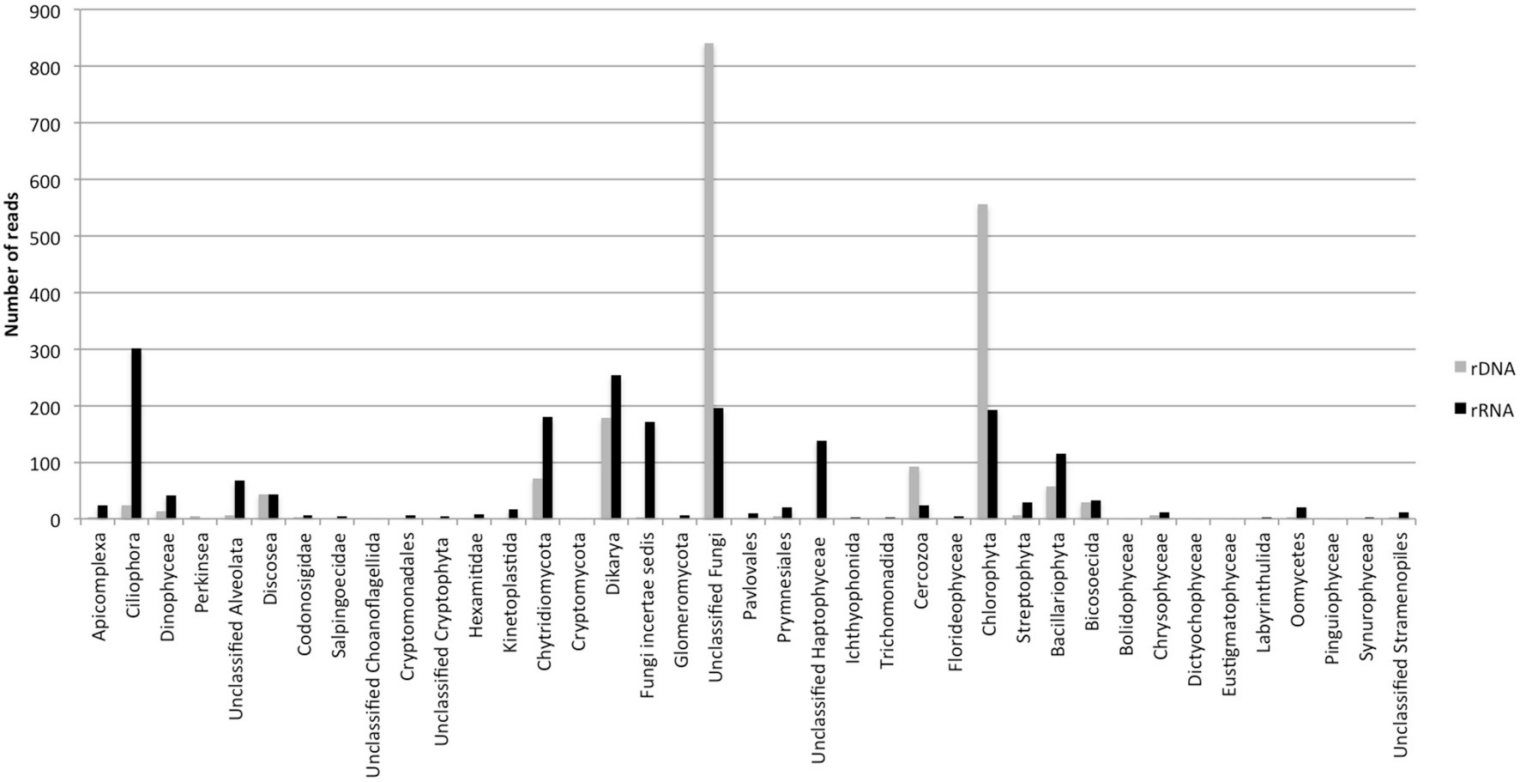
**Numbers of rDNA and rRNA reads in the monimolimnion of lake Pavin (454 dataset from the 05/07/2011)**.

**Figure 5:**
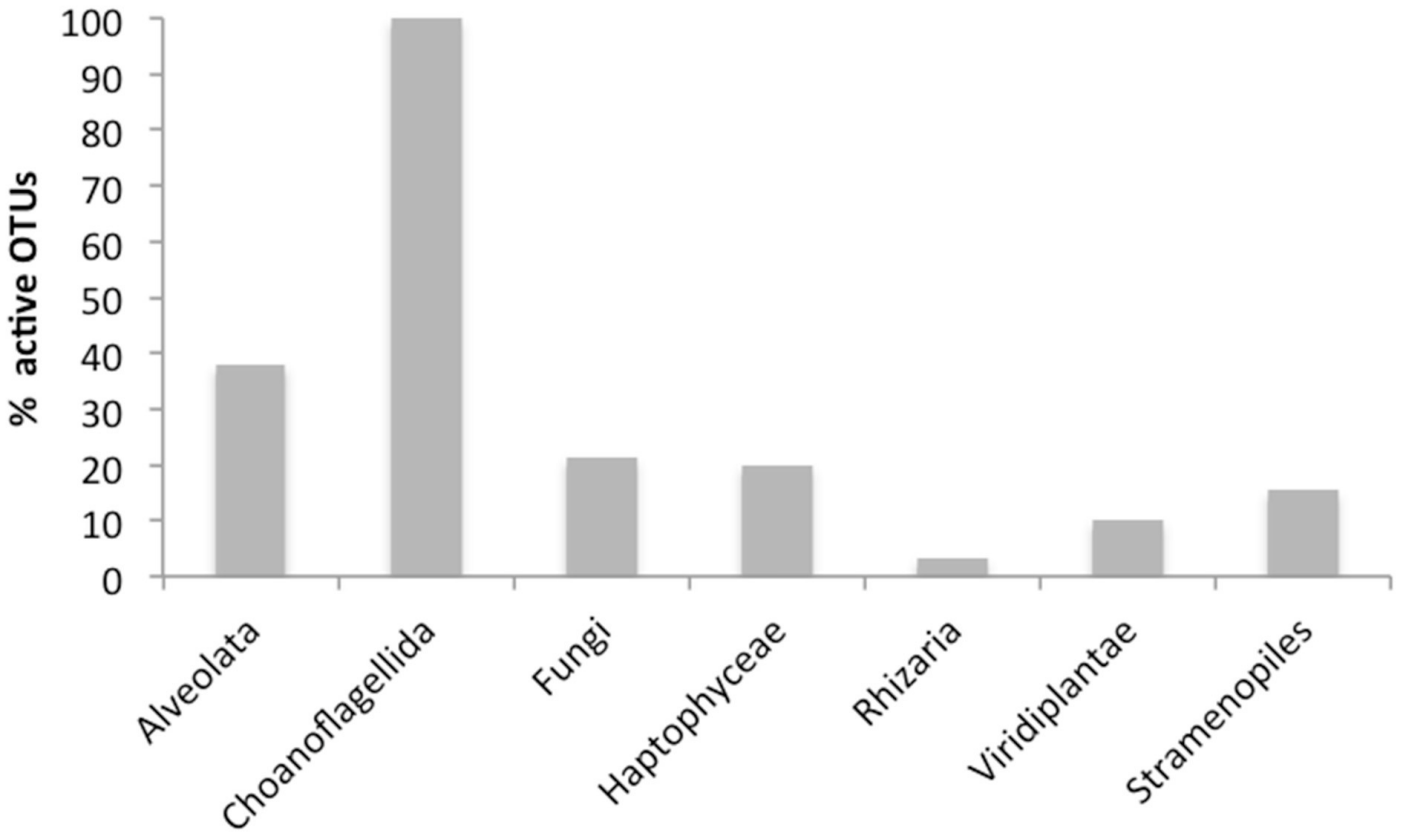
**Percentage of active OTUs (i.e., rRNA:rDNA ratio > 1) in the monimolimnion of lake Pavin (454 dataset from the 05/07/2011)**.

The dynamics of eukaryotic 18S rRNA transcripts in the monimolimnion shows that some eukaryotes are certainly active along the sampling period in the permanent anoxic zone of Lake Pavin. The number of 18S rRNA transcripts increases from March to April (from 16,000 to 26,000 transcripts per mL) and then decreases continuously until July (around 2300 transcripts per mL) (Figure 2). During the summer period, 18S rRNA transcripts numbers reach 6800 copies per mL but this number declines from October to December (<1000 transcripts per mL). The presence of active eukaryotes at 80 m is confirmed by TSA-FISH (which use ribosomal RNA-targeted nucleic acid probes). Indeed we highlighted the recurrent presence of eukaryotes at 80 m along the sampling period, also with maximal abundances in March/April (Supplementary Figure 2). Specifically, we were able to show positive signals with the probes, targeting, Fungi, Chrysophyceae, Chlorophyceae, Haptophyceae, and Cryptophyceae (Figure 3). On one date (05/07/2011), by targeting the 18S rRNA, we found the same dominant phylogenetic groups assessed by 18S rDNA: Viridiplantae, Fungi, and Alveolata (Figure 4). With the exception of Perkinsozoa, Chlorophyta, Cercozoa, and unclassified Fungi, the rRNA sequences are more abundant than rDNA sequences. At the OTU level, the mean rRNA:rDNA ratio computed from each OTU is 2.2. This mean ratio reaches 3.0 when calculated for Viridiplantae and 7.9 for Fungi. 17.9% (419 OTUs) of the OTUs recovered from the monimolimnion are active (i.e., rRNA:rDNA ratio > 1). The relationship between 18S rRNA and rDNA frequency for each OTUs shows that their contribution to the total activity is highly variable (Supplementary Figure 5). Choanoflagellida, Alveolata, Fungi, Haptophyceae, Viridiplantae, and Stramenopiles present the higher percentage of active OTUs (Figure 5). Within the Alveolata active OTUs, only Ciliophora and Dinophyceae are found. The Viridiplantae active OTUs (10% of total OTUs; Figure 5) are mostly represented by Chlorophyta and the Haptophyceae by Prymnesiales whereas active OTUs within Stramenopiles are more diverse being represented by Bicosoecida, Chrysophyceae, and diatoms. It's noteworthy that 20% of the active OTUs found in the monimolimnion are specific to this zone. Half of them are Fungi OTUs (Dikarya and Chytrids), however Choanoflagellida, Viridiplantae (Chlorophyta) and Stramenopiles (Bicosoecida) are also found. These results highlight the fact that microbial eukaryotes, including putative photosynthetic taxa are active in a dark and anoxic zone of a freshwater lake.

## Discussion

Recent eukaryotic diversity surveys based on 18S rRNA reveal an unexpected variety of often divergent lineages in different biotopes. Among these are several extreme environments [e.g., oxygen-depleted sediments, highly acidic freshwater, deep-sea habitats including hydrothermal vents, and Antarctic soils (Dawson and Pace, 2002; Zettler et al., 2002)]. However, eukaryotic community has rarely been studied by NGS in a permanent anoxic zone of a freshwater meromictic lake. Here, we provide new insights into this specific ecosystem, focusing on taxonomic composition of the smallest microbial eukaryotes (size around 5 μm) as well as their activity inferred by 18 S rRNA. It is well known that the general use of rRNA as a reliable indicator of the metabolic state in microbial assemblages has some limitations (Blazewicz et al., 2013). For example, dormant cells can contain a high number of ribosomes. Therefore, in environments that could likely contain dormant cells, which can be the case for the monimolimnion of lake Pavin, employing rRNA to identify current activity can be problematic. However, in the absence of other methodologies to study the metabolic activity of microbial communities, the use of the rRNA index seems to be the best option (Lankiewicz et al., 2015). In addition, to confirm rRNA sequencing results, we used TSA-FISH technique to visualize active cells.

As already shown previously (Lepère et al., 2006; Debroas et al., 2015), microbial eukaryotic community in the oxic mixolimnion of lake Pavin exhibited changes in its taxonomic composition over seasons. However, unexpected changes in the monimolimnion were also recorded, especially when considering a fine taxonomic resolution (OTU level). This could be caused by sedimentation from the top to the bottom of the lake, however, from our dataset, we could not detect any similarities between the OTUs composition at 2 m and 80 m that might suggest only a sedimentation process (Supplementary Figure 3). Moreover, the dynamics of eukaryotic 18S rRNA transcripts in the monimolimnion showed that eukaryotes are globally active along the sampling period but this activity is unstable, with peaks in spring and summer. In addition, the temporal changes detected at 80 m were partially driven by active OTUs specific to the monimolimnion. Even though this zone is not directly affected by atmospheric changes such as temperature and light availability, nutrient (PO_4_, NO_2_, NO_3_, and NH_4_) concentrations change along the sampling period (Hugoni et al., 2015 and Supplementary Table 1). Some authors have suggested the existence of under lake springs (Martin, 1985; Camus et al., 1993), but these entrances are not clearly identified. Considering the volcanic context of lake Pavin, part of these contributions could be gaseous mineral water (Assayag, 2003). The temporal changes of phosphate, ammonia and nitrate concentrations in the anoxic zone of lake Pavin could affect the dynamics of active eukaryotes as previously suggested in a study focusing on archaeal diversity in this zone (Hugoni et al., 2015). Altogether, these results highlight that the anoxic zone of this meromictic lake should not be considered as particularly stable in its microbial composition.

At a low taxonomic resolution there are only moderate differences in the relative proportion of major phylogenetic groups between the mixolimnion and the monimolimnion. Nonetheless, at a finer taxonomic resolution spatial community structure changes are more obvious, with 42% of OTUs being specific to the monimolimnion. Among these OTUs, Fungi are well represented and active according to rRNA analysis. The presence of active Fungi in the anoxic zone is not surprising. Indeed, they are well documented from aquatic anoxic habitats (Luo et al., 2005; Stoeck et al., 2010; Orsi et al., 2012), and recent experiments in the Arabian sea show evidence for fungal anaerobic metabolism (Stief et al., 2014). The recovery of both rRNA and rDNA of anaerobic Fungi suggests that they are metabolically active and might complement bacteria and Archaea in the utilization and recycling of nutrients. Globally, Fungi are capable of adapting to extreme environments since they are found in anoxic ecosystem, extreme acid waters, and on hydrothermal vents. More precisely, some Chytrids OTUs are active and specific to the monimolimnion. Various phylogenetic analyses have shown that anaerobic Chytrids cluster with their aerobic relatives (Li et al., 1993; Hackstein et al., 1999), suggesting that they evolved from aerobic mitochondriate, but lack mitochondria and instead possess hydrogenosomes, organelles that compartmentalize the terminal steps of anaerobic energy metabolism (Hackstein et al., 1999). Dikarya (Ascomycota and Basidiomycota) are also found in the monimolimnion probably acting as saprotrophs. They are related to terrestrial representatives and may derive from the littoral vegetation in the lakes and thus may have been introduced from lake water or pollen grains (Wurzbacher et al., 2014).

Stramenopiles, mainly Chrysophyceae and Bicosoecida represent 14% of the OTUs in the monimolimnion of lake Pavin, some of which can be active (Figure 5). Chrysophyceae and Bicosoecida are frequent and abundant members of oxygen-depleted habitats (Luo et al., 2005; Stock et al., 2009; Behnke et al., 2010; Orsi et al., 2012). A recent marine survey showed that several MAST groups were typical of anoxic systems (Massana et al., 2009). These data further support the view of oxygen as a fundamental driver of microbial eukaryotes community structure. Stramenopiles are known for their involvement in regulating bacterial populations in oxic waters and could have the same ecological role in anoxic waters. Studies such as Fenchel and Finlay (2008) have found that some eukaryotes thrive at specific oxygen concentration, creating their own niche among competitors with similar living and feeding habits. As many species feed on bacteria, low-O_2_ waters could act as a cue for feeding, as bacterial production tends to be elevated at or below the oxycline (Fenchel et al., 1990; Fenchel and Finlay, 2008). However, Oikonomou et al. (2014b) showed that grazing in the anoxic monimolimnion of a freshwater meromictic lake was negligible with a low prokaryote turn over rate.

Active OTUs of Dinoflagellates and Ciliates dominate the Alveolata (which is one of the most active group in the monimolimnion) and are present throughout the sampling period. The biological versatility of Dinoflagellates makes them a very successful entity in freshwater ecosystems (Taylor et al., 2008) and can adapt to low light conditions (Jakobsen et al., 2000). However, they might be under the cyst form to survive in anoxic waters (McCarthy et al., 2013). Even though ciliates tend to prefer oxic/anoxic boundary layers in aquatic habitat (Fenchel and Finlay, 2008), they are also very successful in low-oxygen conditions or anoxia (Fenchel and Finlay, 1995). In these environments, Ciliates grazing can impact a large proportion of anaerobic picoplankton production (Sàcca et al., 2009). Ciliates can also form cysts, a reversible state of reduced metabolic activity, when the conditions are less favorable (e.g., low temperature) (Lennon and Jones, 2011). Twenty-two percent Ciliates OTUs are specific to the monimolimnion of lake Pavin and are generally < 85% similar to deposited sequences of described taxa. The closest reference sequences for these OTUs are found in suboxic and sulfidic marine waters (Wylezich and Jürgens, 2011).

Surprisingly, with 30% of the OTUs, Viridiplantae is the dominant phylum in the monimolimnion of lake Pavin. Anoxic compartments of stratified water columns are generally dominated by ciliates, and Viridiplantae usually represents < 5% of OTUs (Alexander et al., 2009; Behnke et al., 2010; Charvet et al., 2012; Oikonomou et al., 2014a). Even though rRNA reads are less abundant than rDNA reads, at the OTU level, the Viridiplantae, mostly represented by the Chlorophyta division, present 10% of active OTUs with 3 active OTUs specific to the monimolimnion. Their activity is also confirmed by TSA-FISH analyses, which targets ribosomal RNA (Figure 4). In addition, flow cytometry showed pigmented microbial eukaryotes (4800 cell.mL^−1^) at 80 m depth (Supplementary Figure 6). The presence of Chlorophyta is not unusual in dark and anoxic conditions. Some members of the Chlorophyta are indeed metabolically flexible and can turn to anaerobic metabolism after exposure to anoxia and darkness (Atteia et al., 2013). Among the active Chlorophyta discovered at 80 m in lake Pavin, OTUs are affiliated to Chlorophyceae (*Chlamydomonas, Mychonastes*, and *Monoraphidium*), and Trebouxiophyceae (*Chlorella*). *Chlamydomonas reinhardii* and *Chlorella* have the most extensive set of fermentative enzymes reported so far which allow this unicellular organisms to tolerate and colonize anoxic environments, especially the monimolimnia of meromictic lakes (Klaveness and Løvhøiden, 2007; Oikonomou et al., 2014b). Recently, the discovery of the importance of phagotrophy to algal nutrition has highlighted that algae can be consumers as well as primary producers within the microbial loop (Hartmann et al., 2012, 2013; Unrein et al., 2014). However, no evidence of phagotrophy for Chlorophyceae has been shown so far (Unrein et al., 2014). In addition, Oikonomou et al. (2014b) showed that pigmented flagellates accounted for 70% of total grazing in the mixolimnion but suggested that this grazing was negligible in the anoxic monimolimnion. Chlorophyta may also escape from the predation they experience in the photic zones of lakes (Arvola et al., 1992). Within active pigmented eukaryotes, members of the Haptophytes are also present in the monimolimnion of lake Pavin. Although they mostly occur in marine waters, they can also be found in freshwater lakes (Lefranc et al., 2005; Lepère et al., 2010; Simon et al., 2013). However, Haptophytes have not been found in other anoxic layer of meromictic lake (Charvet et al., 2012; Oikonomou et al., 2014a), but have been reported in an anoxic fjord in very low proportion (Behnke et al., 2010). In the lake Pavin monimolimnion, they represent 1.6% of total OTUs and 20% of these OTUs are active. These active OTUs are affiliated to the Prymnesiales. Contrary to some Chlorophyta, they are not known for anaerobic metabolism. Among active Prymnesiales, OTUs affiliated with *Prymnesium* and *Chrysochromulina* are the most frequently encountered. Prymnesium shows tolerance for an extremely broad range of salinities and can produce toxins. Chrysochromulina occurs worlwilde in polar as well as warmer waters. This genus has a very long haptonema that functions in prey capture (Hansen, 1998). Experimental studies suggest that phagotrophy may be a method of acquiring phospholipids from bacteria as a source of phosphate (Jones et al., 1994). Many species are also able to take up dissolved organic carbon, which is thought to contribute to their survival in low light environments.

In conclusion, this study has revealed a high genetic diversity of microbial eukaryotes in anoxic waters of a meromictic lake and allowed the discrimination of active and inactive components. In the anoxic zone, heterotrophic microorganisms (such as Fungi and Alveolata) are the most highly active, but more surprisingly, several taxonomic groups of pigmented microbial eukaryotes are also present and active (as revealed by DNA/RNA ratio). The low abundance of microbial eukaryote predators in such waters could provide them a refuge. Some data suggest that microbial eukaryotes grazing (including pigmented ones) in anoxic waters is lower than in oxygenated ones with a notable shift in the microbial carbon pathway from typical grazing to parasitism, which would explain the high contribution of fungal parasites such as Chytrids. The presence of active microbial eukaryotes could also be explained by original metabolic capabilities. Explorations of biological diversity under some of the most extreme environmental conditions will likely lead to the discovery of novel microorganisms. It is therefore absolutely critical to isolate and genetically characterize these organisms.

## Author contributions

Conceived and designed the experiments: DD, ID. Performed the experiments: AV, MH, ID. Analyzed the data: CL, DD, ID. Contributed reagents/materials/analysis tools: DD, ID. Wrote the paper: CL, DD, ID, MH, AV.

### Conflict of interest statement

The authors declare that the research was conducted in the absence of any commercial or financial relationships that could be construed as a potential conflict of interest.

## Footnotes

^1^http://datadryad.org/resource/doi:10.5061/dryad.5mv53

## Supplementary material

Supplementary Table 1 **Environmental parameters (pH, temperature and oxygen, phosphate, nitrate and ammonia concentrations) in the monimolimnion of lake Pavin (Hugoni et al., 2015)**.

Supplementary Table 2 **Identity of OTUs with Silva database determined by Blast analysis in the mixolimnion and monimolimnion**.

Supplementary Table 3 **Taxonomic composition of microbial eukaryotes in the mixolimnion and monimolimnion (rDNA OTU distributions, average over the study period)**.

Supplementary Figure 1 **Flow chart describing the bioinformatics pipeline**.

Supplementary Figure 2 **Dynamics of microbial eukaryotes abundances in the mixolimnion and monimolimnion obtained with the oligonucleotide probe EUK1209**.

Supplementary Figure 3 **Dendogram representing the hierarchical cluster analysis (OTUs level) between 2 (Red) and 80 m (Green)**.

Supplementary Figure 4 **Dynamics of rDNA OTUs in the monimolimnion of lake Pavin**.

Supplementary Figure 5 **Relationships between 18S rRNA and 18S rDNA frequencies of eukaryotic OTUs defined in the dataset**. The dotted line is the 1:1 line.

Supplementary Figure 6 **Cytogram obtained from monimolimnion samples**. Cytogram correspond to phycoerythrin orange fluorescence (FL2) vs. chlorophyll red fluorescence (FL3), both in arbitrary units. Ellipses emphasize two photosynthetic populations based on multiparameter gating: photosynthetic picoeukaryotes and picocyanobacteria.
