# Supplementary material for: Diversity and Dynamics of Active Small Microbial Eukaryotes in the Anoxic Zone of a Freshwater Meromictic Lake (Pavin, France)
Source: Front Microbiol. 2016 Feb 10;7:130. doi: 10.3389/fmicb.2016.00130 (PMC4748746; doi:10.3389/fmicb.2016.00130)
Supplement: Supplementary Table 1 — Environmental parameters (pH, temperature and oxygen, phosphate, nitrate and ammonia concentrations) in the monimolimnion of lake Pavin (Hugoni et al., 2015). [file Table1.PDF]

Depth, m	Sampling Date	pH	Temp., °C	Oxygen, $mg.L^{-1}$	PO_4^{3-}, $mgP.L^{-1}$	NO_2^-, $mgN.L^{-1}$	NO_3^-, $mgN.L^{-1}$	NH_4^+, $mgN.L^{-1}$
80	21-Mar-11	ND	5.1	0.36	2.79	0.08	0	71.39
	13-Apr-11	ND	5	0.41	0	0.05	0	54.61
	26-Apr-11	ND	4.2	0.55	0	0.12	0.27	98.46
	10-May-11	6.35	4.5	0.41	40.20	0.11	0.34	87.11
	6-Jun-11	5.98	ND	ND	3.33	0.18	0	9.03
	5-Jul-11	6.18	ND	ND	4.59	0.15	0.16	10.68
	23-Aug-11	6.27	ND	ND	4.54	0.14	0	9.22
	6-Sep-11	7.09	ND	ND	4.4	0.13	0.24	5.29
	4-Oct-11	6.12	ND	ND	6.1	0.15	0	9.13
	18-Oct-11	6.15	ND	ND	4.29	0.17	0.87	7.20
	15-Nov-11	5.98	ND	ND	4.05	0.16	0	6.57
	5-Dec-11	5.87	ND	ND	2.19	0.08	0	5.77
