# Supplementary material for: Diversity and Dynamics of Active Small Microbial Eukaryotes in the Anoxic Zone of a Freshwater Meromictic Lake (Pavin, France)
Source: Front Microbiol. 2016 Feb 10;7:130. doi: 10.3389/fmicb.2016.00130 (PMC4748746; doi:10.3389/fmicb.2016.00130)
Supplement: Supplementary Table 2 — Identity of OTUs with Silva database determined by Blast analysis in the mixolimnion and monimolimnion. [file Table2.PDF]

Similarity (%) to the closest reference sequence (Silva database)	OTUs 2 m	OTUs 80m
95-100	3786	3636
85-94.9	3028	3753
<85	69	349
