# Supplementary material for: Diversity and Dynamics of Active Small Microbial Eukaryotes in the Anoxic Zone of a Freshwater Meromictic Lake (Pavin, France)
Source: Front Microbiol. 2016 Feb 10;7:130. doi: 10.3389/fmicb.2016.00130 (PMC4748746; doi:10.3389/fmicb.2016.00130)
Supplement: Supplementary Table 3 — Taxonomic composition of microbial eukaryotes in the mixolimnion and monimolimnion (rDNA OTU distributions, average over the study period). [file Table3.PDF]

Taxonomic affiliation		% rDNA OTUs 2m	80m
Alveolata	Apicomplexa	2.67	1.68
	Ciliophora	6.59	3.70
	Dinophyceae	6.67	4.95
	Perkinsea	0.17	0.48
	Unclassified Alveolata	2.69	1.47
Amoebozoa	Centramoebida	3.11	7.05
Choanoflagellida	Codonosigidae	0.09	0.22
	Unclassified Choanoflagellida	0.04	0.13
Cryptophyta	Cryptomonadales	0.39	0.13
	Unclassified Cryptophyta	0.30	0.02
Diplomonadida	Hexamitidae	0.17	0.43
Euglenozoa	Kinetoplastida	4.05	1.80
Fungi	Chytridiomycota	6.57	3.98
	Cryptomycota	0.51	0.10
	Dikarya	11.04	14.36
	Fungi incertae sedis	3.72	1.14
	Glomeromycota	0.01	0.22
	Nowakowskiella clade	0.29	0.05
	Tremellales	0.10	0.13
	Unclassified Fungi	1.32	5.99
Haptophyceae	Pavlovales	0.13	0.01
	Prymnesiales	1.22	0.67
	Unclassified Haptophyceae	1.87	0.88
Ichthyosporea	Ichthyophonida	0.10	0.15
Parabasalia	Trichomonadida	1.23	1.38
Rhizaria	Cercozoa	1.80	4.15
	Unclassified Cercozoa	0.67	0.09
Rhodophyta	Florideophyceae	0.13	0.24
Viridiplantae	Chlorophyta	21.85	29.19
	Streptophyta	0.46	1.14
Stramenopiles	Bacillariophyta	7.90	8.73
	Bicosoecida	7.00	2.44
	Chrysophyceae	2.57	1.07
	Dictyochophyceae	0.30	0.13
	Eustigmatophyceae	0.04	0.19
	Labyrinthulida	0.26	0.08
	Oomycetes	0.57	0.45
	PX clade	0.06	0.02
	Synurophyceae	0.48	0.26
	Unclassified Stramenopiles	0.78	0.74
