# Supplementary material for: Diversity and Dynamics of Active Small Microbial Eukaryotes in the Anoxic Zone of a Freshwater Meromictic Lake (Pavin, France)
Source: Front Microbiol. 2016 Feb 10;7:130. doi: 10.3389/fmicb.2016.00130 (PMC4748746; doi:10.3389/fmicb.2016.00130)
Supplement: Supplementary Figure 1 — Flow chart describing the bioinformatics pipeline. [file Image1.PDF]

Supporting information Figure 1. Flow chart describing the pipeline PANAM

Taib N, Mangot J-F, Domaizon I, Bronner G, Debroas D (2013) Phylogenetic Affiliation of SSU rRNA Genes Generated by Massively Parallel Sequencing: New Insights into the Freshwater Protist Diversity. PLoS ONE, 8, e58950.

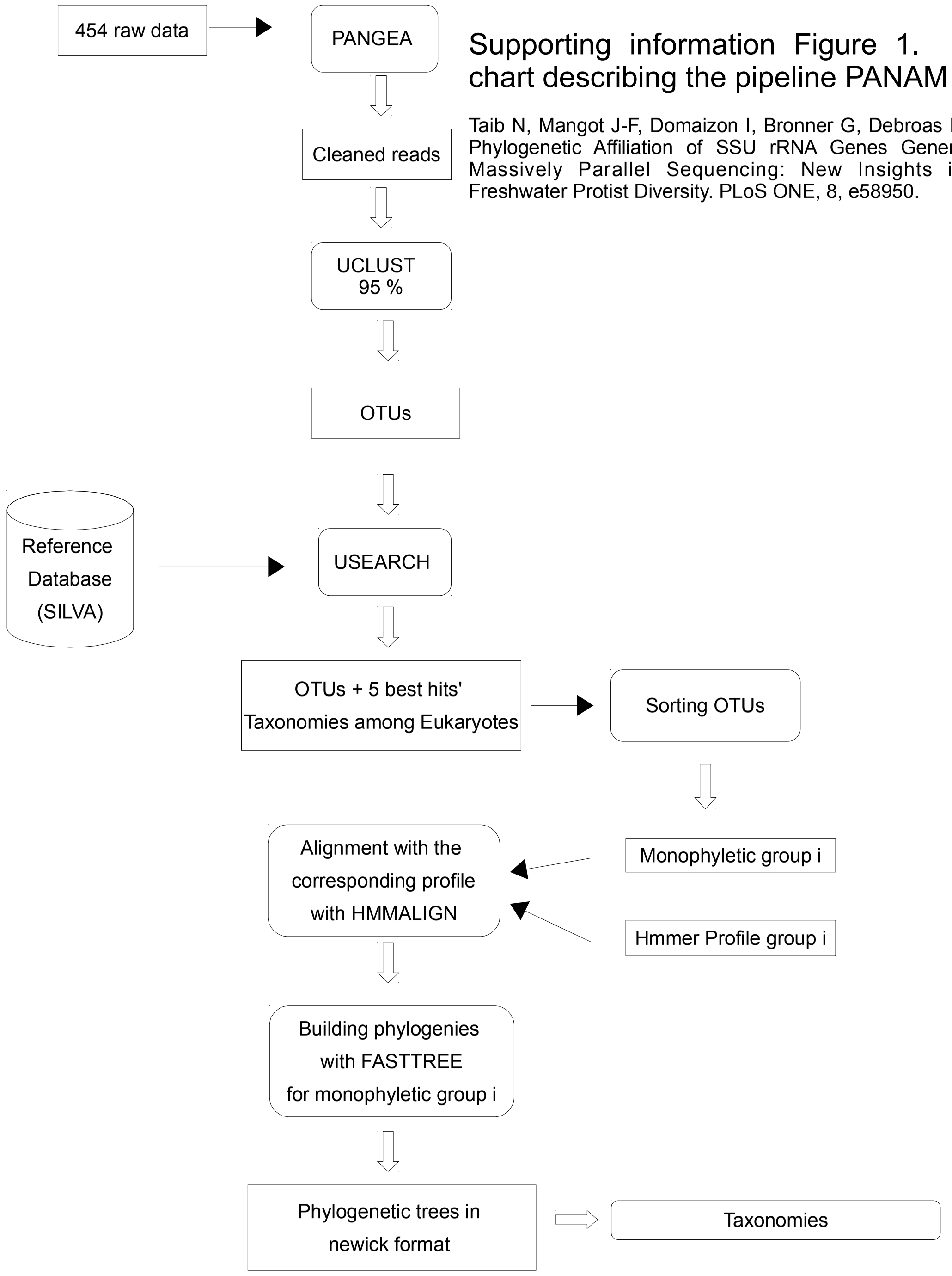

http://code.google.com/p/panam-phylogenetic-annotation /downloads /list

1- Cleaning procedure: short sequences and sequences with low-quality scores are removed and only sequences with a primer match percentage above a defined threshold are selected using Fuznuc .

Giongo A, Crabb DB, Davis-Richardson AG, Chauliac D, Mobberley JM, et al. (2010) PANGEA: pipeline for analysis of next generation amplicons. ISME J 4: 852–861.

2- clustering reads into OTUs with a cut-off of 95 %

Edgar RC (2010) Search and Clustering Orders of Magnitude Faster Than BLAST. Bioinformatics 26: 2460–2461.

3- OTUs are compared against the reference database. The query OTUs are sorted according to the taxonomy of their best hits among eukaryotes, whatever their similarity score. Several files are generated, each containing the reads and their 5 best hits, assigned to one of the 25 specific monophyletic groups among the Eukaryota

Edgar RC (2010) Search and Clustering Orders of Magnitude Faster Than BLAST. Bioinformatics 26: 2460–2461.

4- After reads have been assigned to phyletic groups, they are aligned to the reference sequences of the corresponding profile alignment for that group using hmmalign from the HMMER package.

Eddy SR (1998) Profile hidden Markov models. Bioinformatics 14: 755–763.

5- Using FastTree, a bootstrapped phylogenetic tree (100 iterations) is built for each phyletic profile, including OTUs associated with their 5 best hits and the reference sequences in the profile.

Price MN, Dehal PS, Arkin AP (2010) FastTree 2 – Approximately Maximum- Likelihood Trees for Large Alignments. PLoS ONE 5: e9490.

6- Two methods for taxonomy assessment are implemented: lowest common ancestor (LCA) and nearest neighbor (NN).

Kembel SW, Cowan PD, Helmus MR et al. (2010) Picante: R tools for integrating phylogenies and ecology. Bioinformatics, 26, 1463–1464.
Paradis E, Claude J, Strimmer K (2004) APE: Analyses of Phylogenetics and Evolution in R language. Bioinformatics, 20, 289–290.
