# Supplementary figures and images for: Diversity and Dynamics of Active Small Microbial Eukaryotes in the Anoxic Zone of a Freshwater Meromictic Lake (Pavin, France)
Source: Front Microbiol. 2016 Feb 10;7:130. doi: 10.3389/fmicb.2016.00130 (PMC4748746; doi:10.3389/fmicb.2016.00130)

Eukaryotes abundance in cell ml<sup>-1</sup>

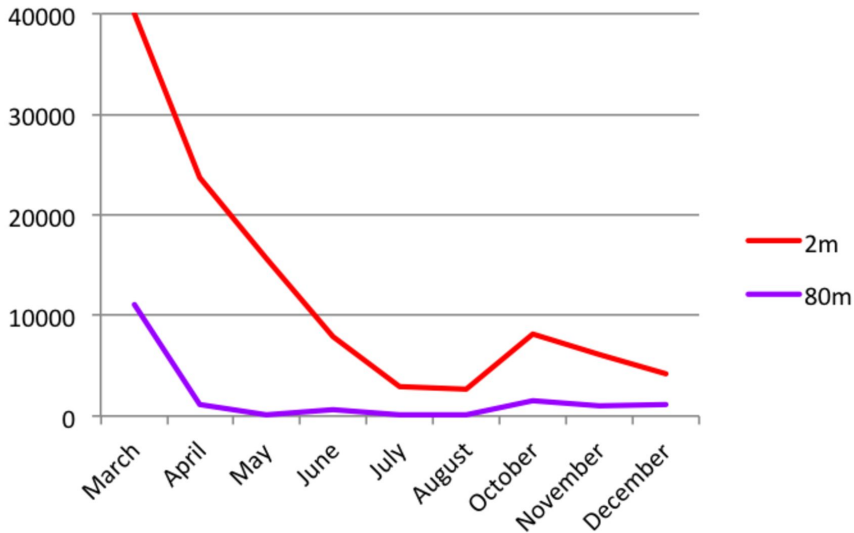

Supplement: Supplementary Figure 2 — Dynamics of microbial eukaryotes abundances in the mixolimnion and monimolimnion obtained with the oligonucleotide probe EUK1209. [file Image2.PDF]

Dendrogram (hierarchical clustering)

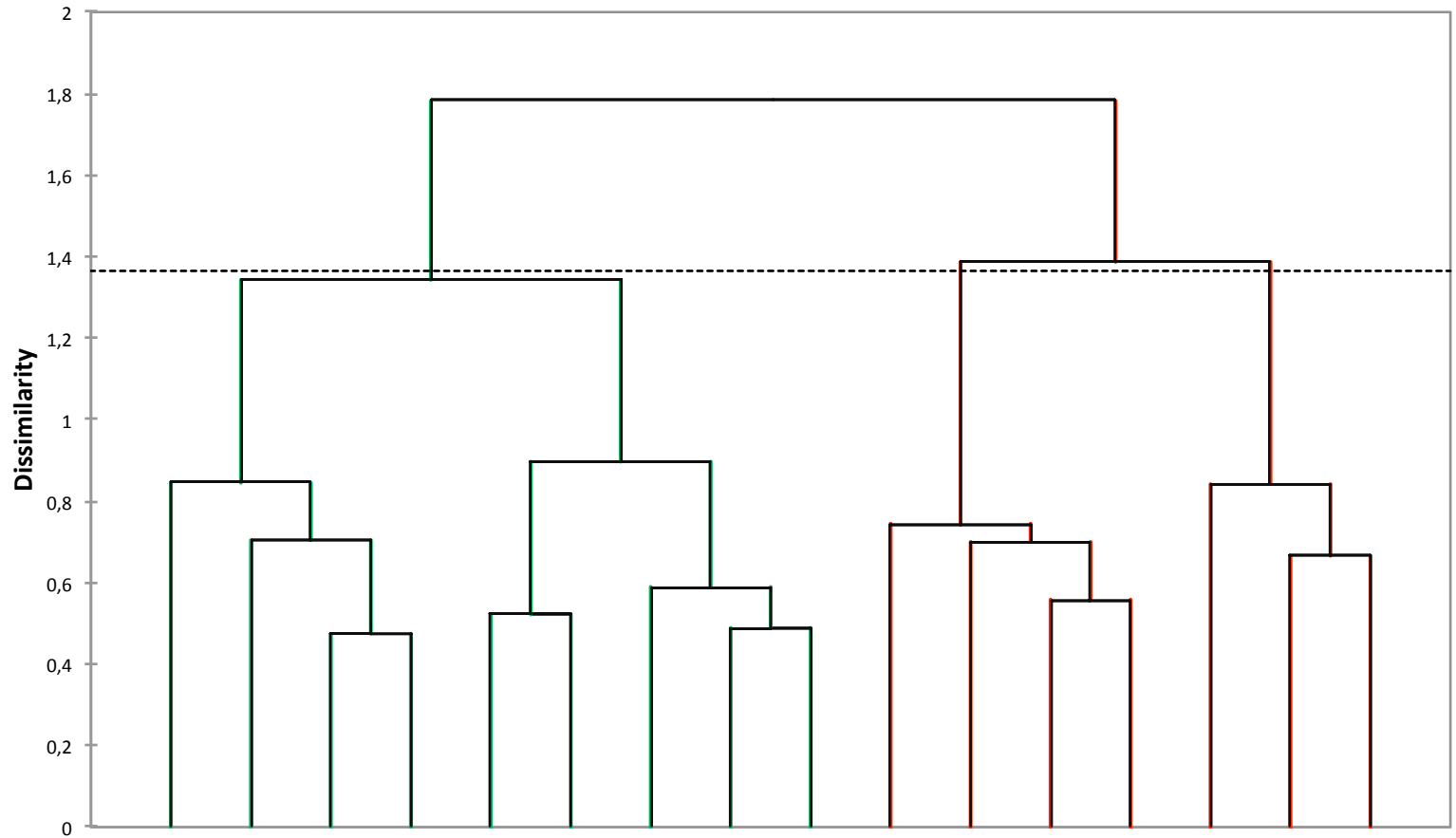

Supplement: Supplementary Figure 3 — Dendogram representing the hierarchical cluster analysis (OTUs level) between 2 (Red) and 80 m (Green). [file Image3.PDF]

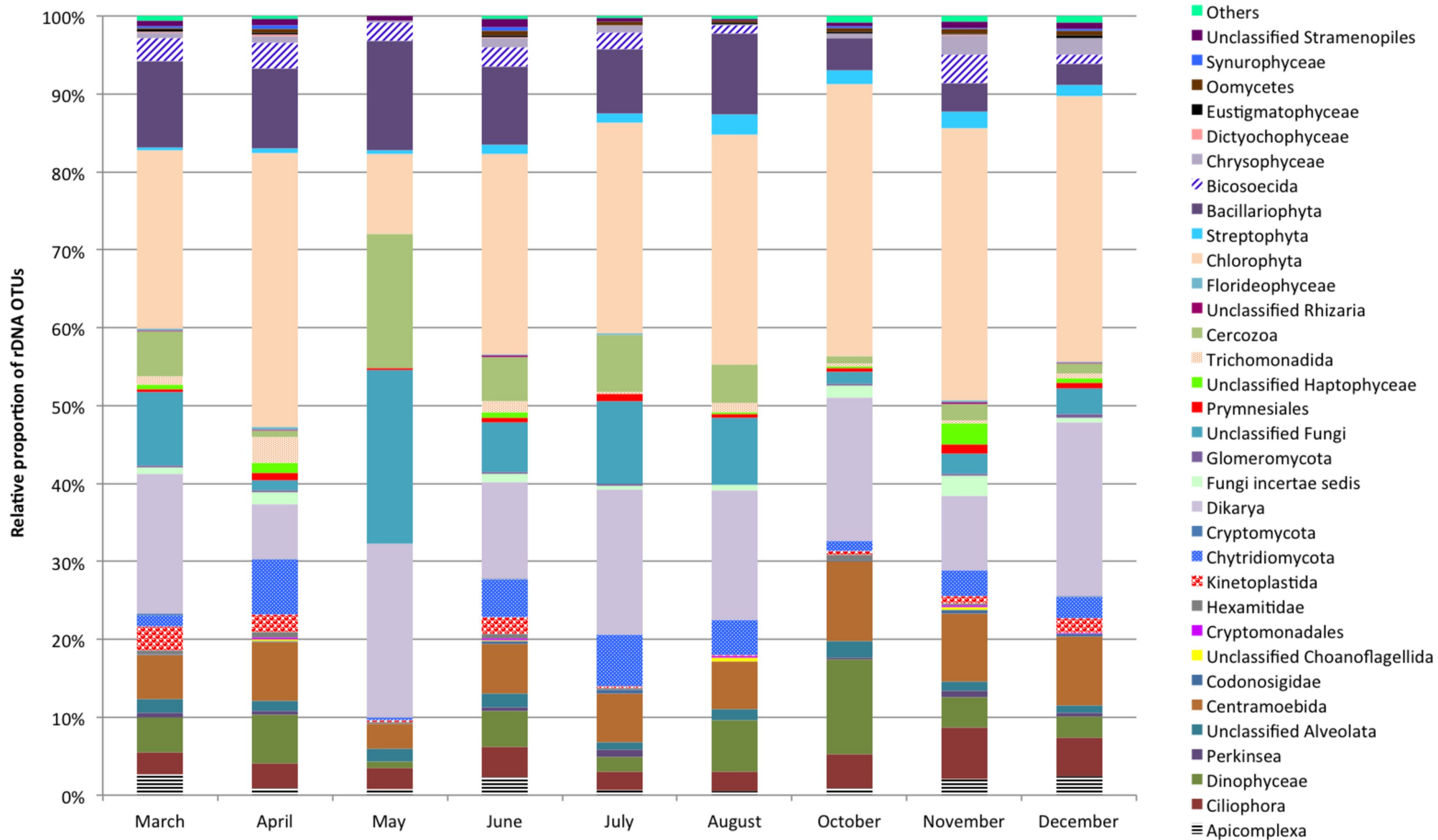

Supplement: Supplementary Figure 4 — Dynamics of rDNA OTUs in the monimolimnion of lake Pavin. [file Image4.PDF]

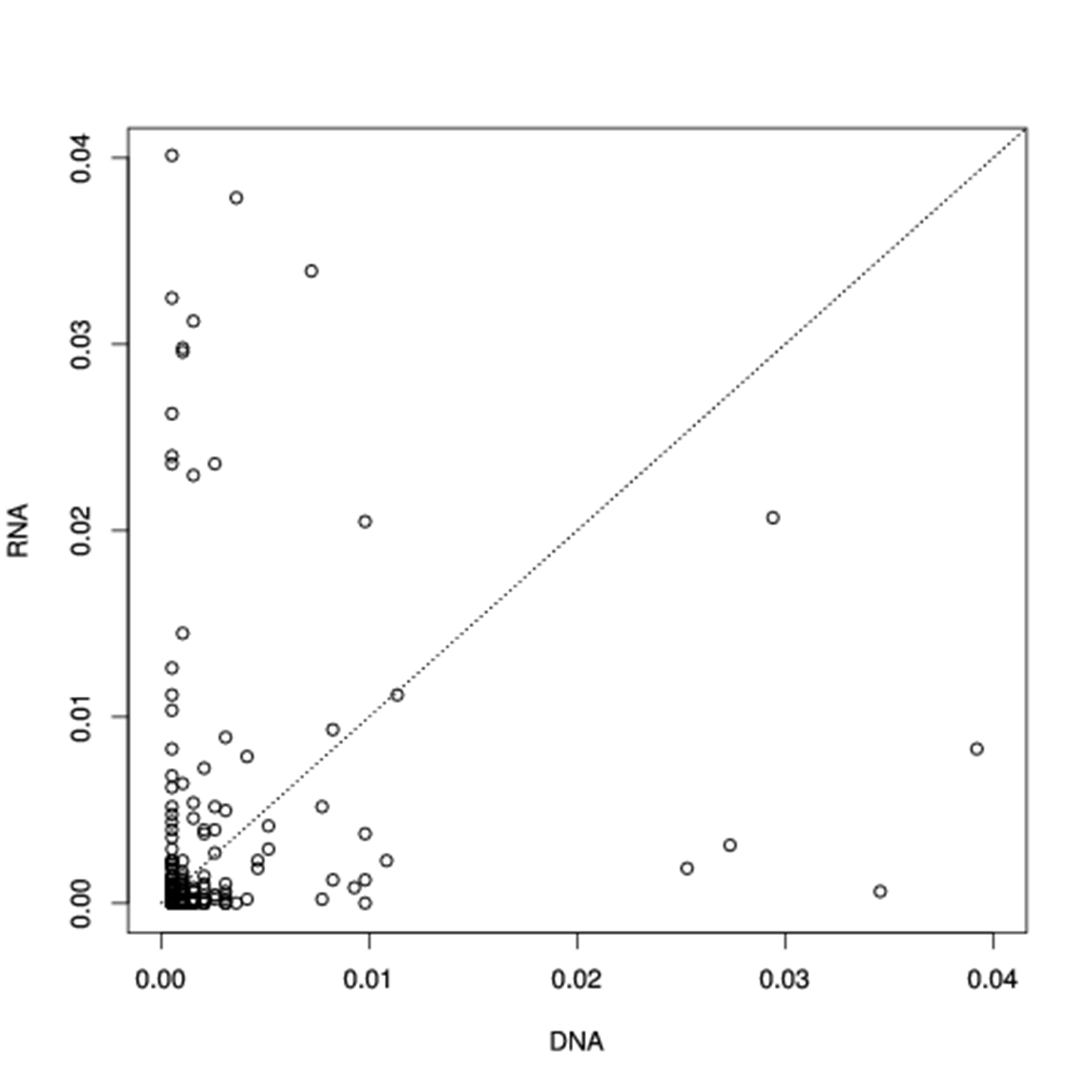

Supplement: Supplementary Figure 5 — Relationships between 18S rRNA and 18S rDNA frequencies of eukaryotic OTUs defined in the dataset. The dotted line is the 1:1 line. [file Image5.TIFF]

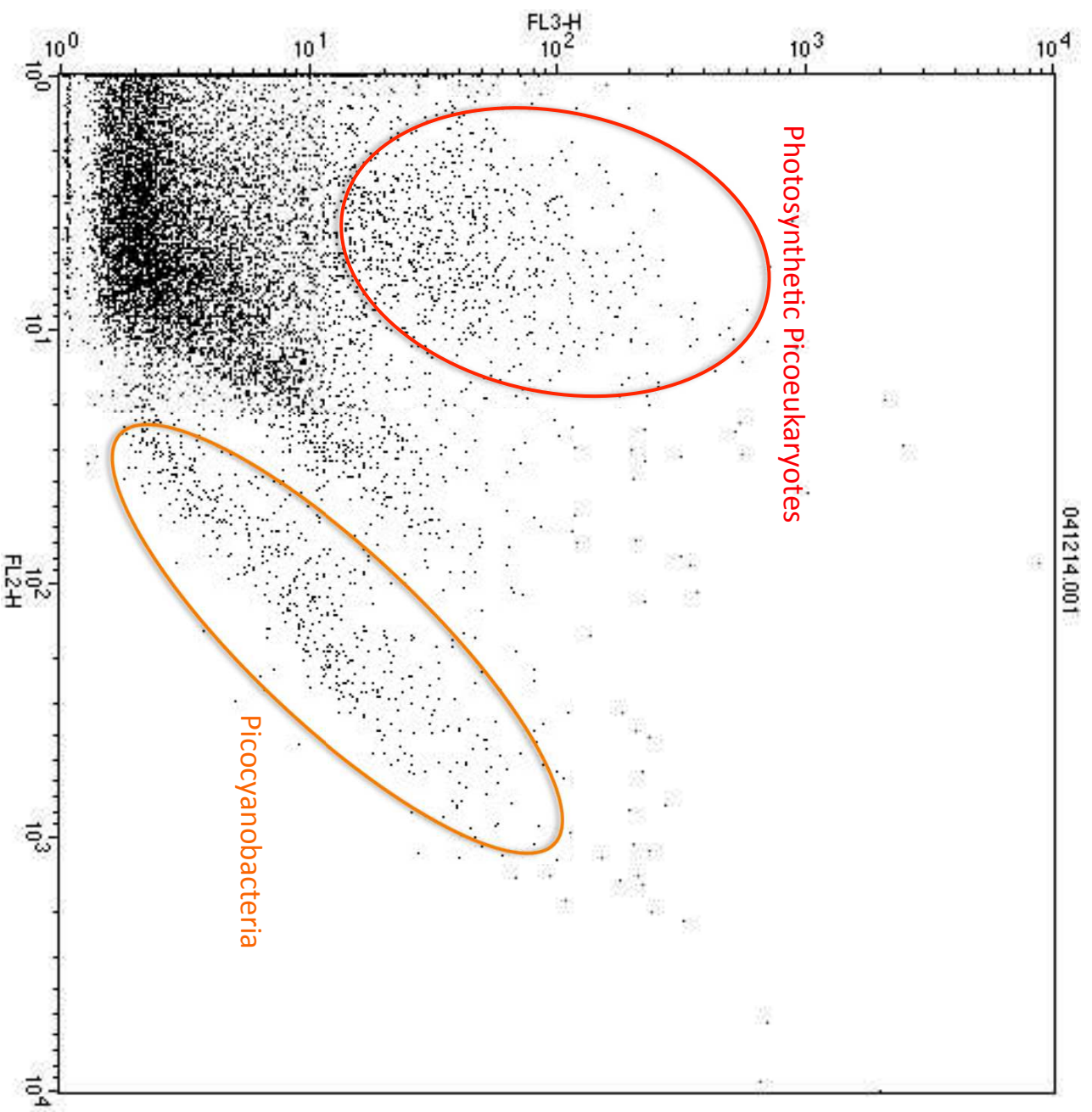

Supplement: Supplementary Figure 6 — Cytogram obtained from monimolimnion samples. Cytogram correspond to phycoerythrin orange fluorescence (FL2) vs. chlorophyll red fluorescence (FL3), both in arbitrary units. Ellipses emphasize two photosynthetic populations based on multiparameter gating: photosynthetic picoeukaryotes and picocyanobacteria. [file Image6.PDF]
